# Supplementary material for: Involvement of a Cluster of Basic Amino Acids in Phosphorylation-Dependent Functional Repression of the Ceramide Transport Protein CERT
Source: Int J Mol Sci. 2022 Aug 2;23(15):8576. doi: 10.3390/ijms23158576 (PMC9368931; doi:10.3390/ijms23158576)
Supplement: Supplementary file 1 [file ijms-23-08576-s001.zip › ijms-1800863-supplementary.pdf]

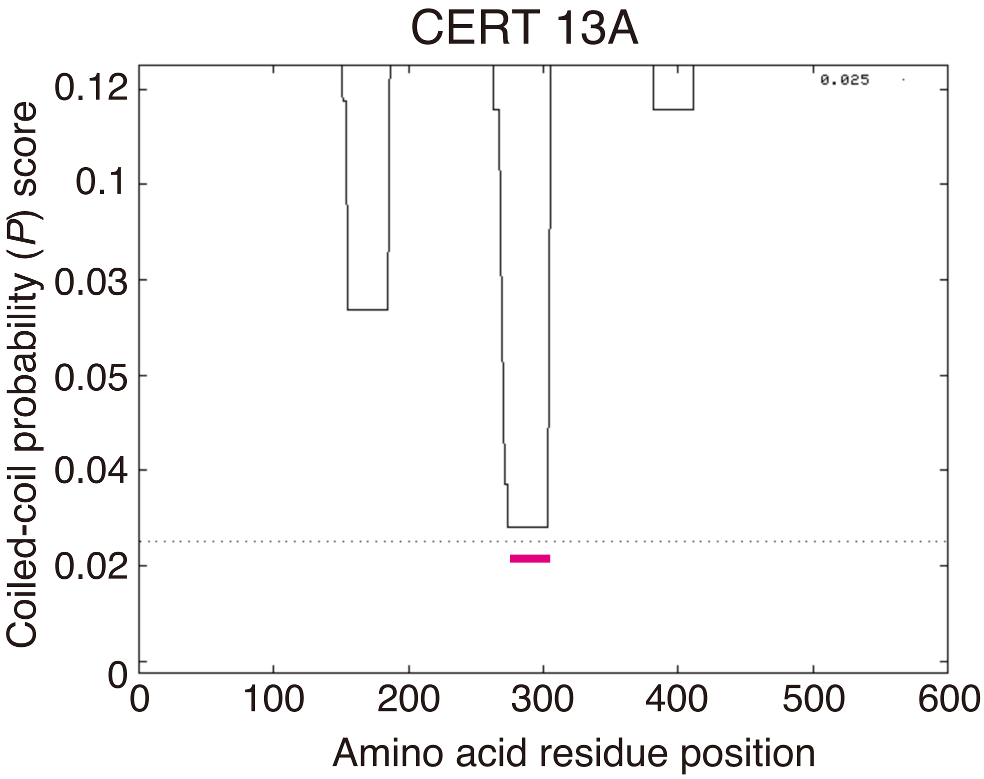

Figure S1. Prediction of CCR in CERT 13A. CERT 13A was expected to have a lower probability of coiled-coil formation than wild-type CERT (Figure 1B). The Paircoil2 program [20] was used to make predictions.

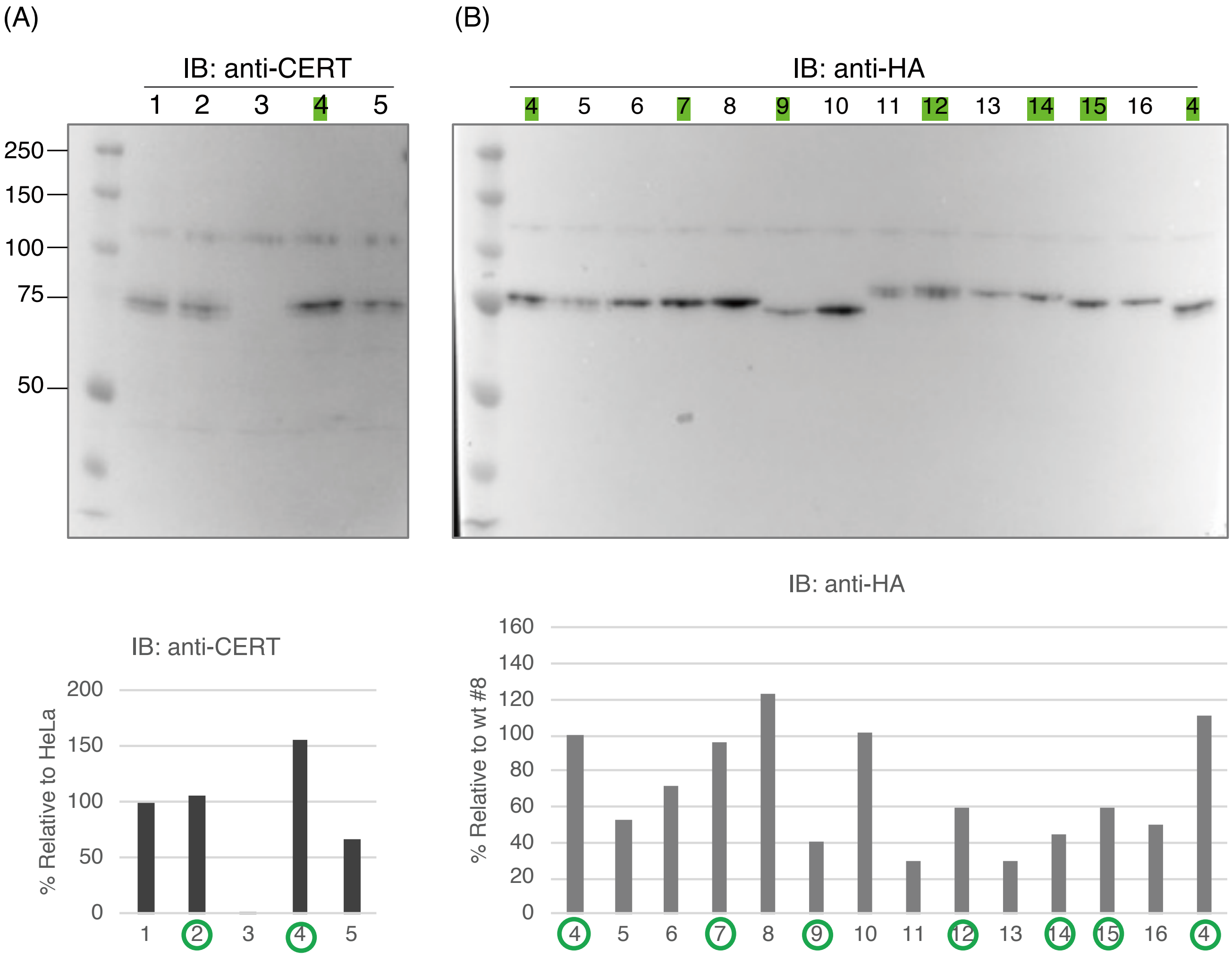

1. HeLa
2. HeLa mCat
3. HeLa mCat ΔCERT
4. HeLa mCat ΔCERT #14 shCERTv1 #1/wt #8
5. HeLa mCat ΔCERT #14 shCERTv1 #1/wt #27
6. HeLa mCat ΔCERT #14 shCERTv1 #1/10E #5
7. HeLa mCat ΔCERT #14 shCERTv1 #1/10E #6
8. HeLa mCat ΔCERT #4 shCERTv1 #1 / 10E #7
9. HeLa mCat ΔCERT #14 shCERTv1 #1/S132A #2
10. HeLa mCat ΔCERT #14 shCERTv1 #1/S132A #8
11. HeLa mCat ΔCERT #14 shCERTv1 #1/13A #3
12. HeLa mCat ΔCERT #14 shCERTv1 #1/13A #6
13. HeLa mCat ΔCERT #14 shCERTv1 #1/10E/13A #4
14. HeLa mCat ΔCERT #14 shCERTv1 #1/10E/13A #7
15. HeLa mCat ΔCERT #14 shCERTv1 #1/S132A/13A #4
16. HeLa mCat ΔCERT #14 shCERTv1 #1/S132A/13A #10

Figure S2. Selection of HeLa CERT KO/shCERT clones stably expressing WT or mutant HA-CERT at levels comparable to endogenous CERT. To suppress the expression level of HA-CERT to the level of endogenous CERT, HeLa CERT KO cells stably transfected with shCERT (CERT KO/shCERT) were used as the parent strain. (A) The expression level of HA-CERT WT clones was compared with the endogenous CERT expression level in the parental HeLa cells by western blotting analysis using anti-CERT antibody. (B) The expression level of HA-CERT mutants was compared with the expression level of HA-CERT WT by western blotting analysis using anti-HA antibody. The lower bar-charts represent the band intensity quantified from the western blotting analysis images (upper panel). The clones highlighted in green were used for immunofluorescence detection of HA-CERT and measurement of SM synthesis. Western blotting images of panel (A) and (B) are uncropped.

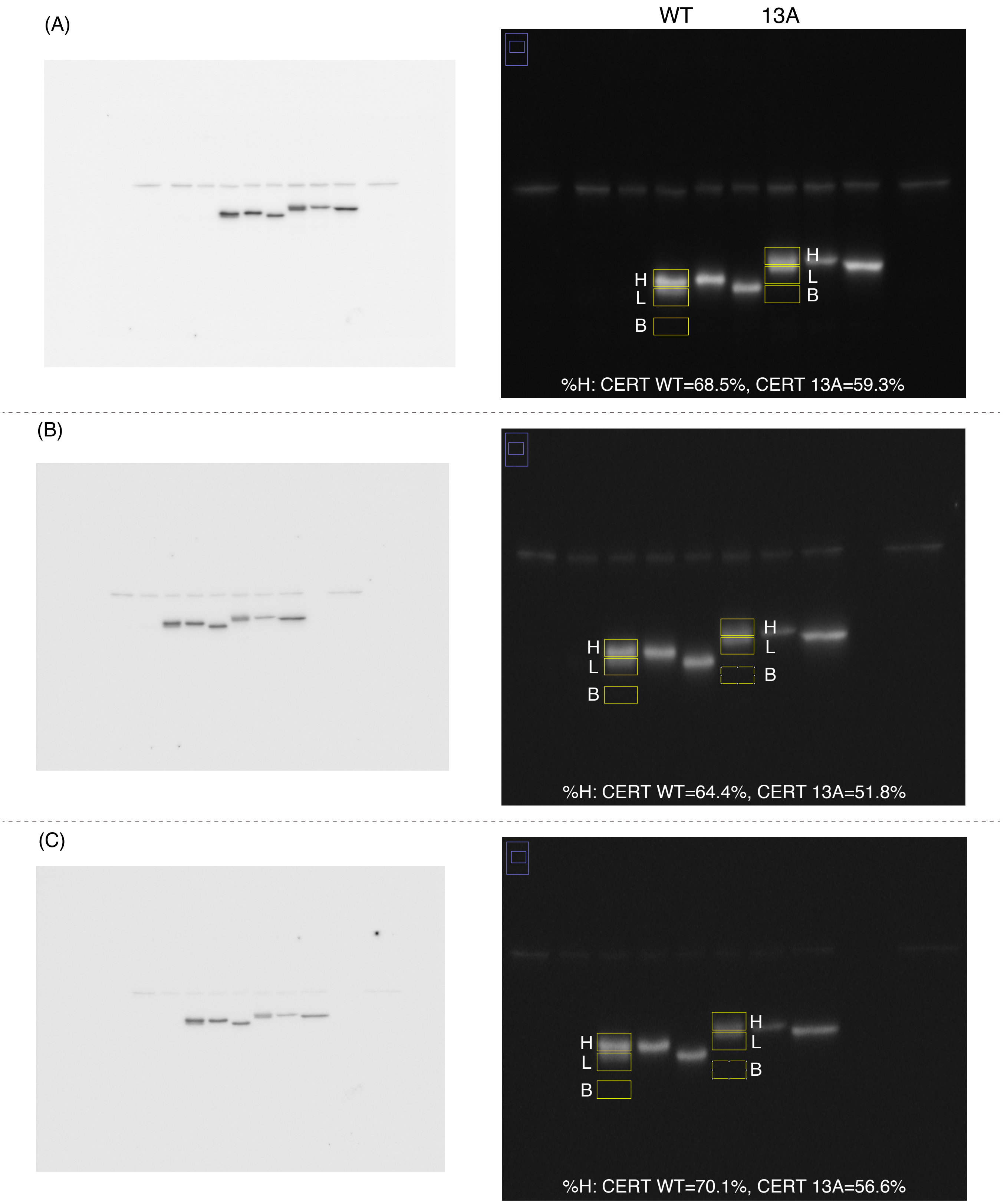

Figure S3. Distribution of the hyperphosphorylated form in CERT WT and 13A. Uncropped images from three independent western blotting analysis experiments (left images in panels A, B, and C) were magnified two-fold in the longitudinal direction (right images in panels A, B, and C), and the signal intensities of regions of interest (ROI) were quantified using ImageJ software [24]: H, L, and B represent the ROI corresponding to the hyperphosphorylated forms of CERTs, de-/hypo-phosphorylated forms of CERTs, and their blank regions, respectively. The percentage distribution values of the hyperphosphorylated form (%H) were calculated for each CERT WT and CERT 13A construct, using the formula:

%H = ([ROI H] — [ROI B]) × 100 /{([ROI H] — [ROI B]) + ([ROI L] — [ROI B])}

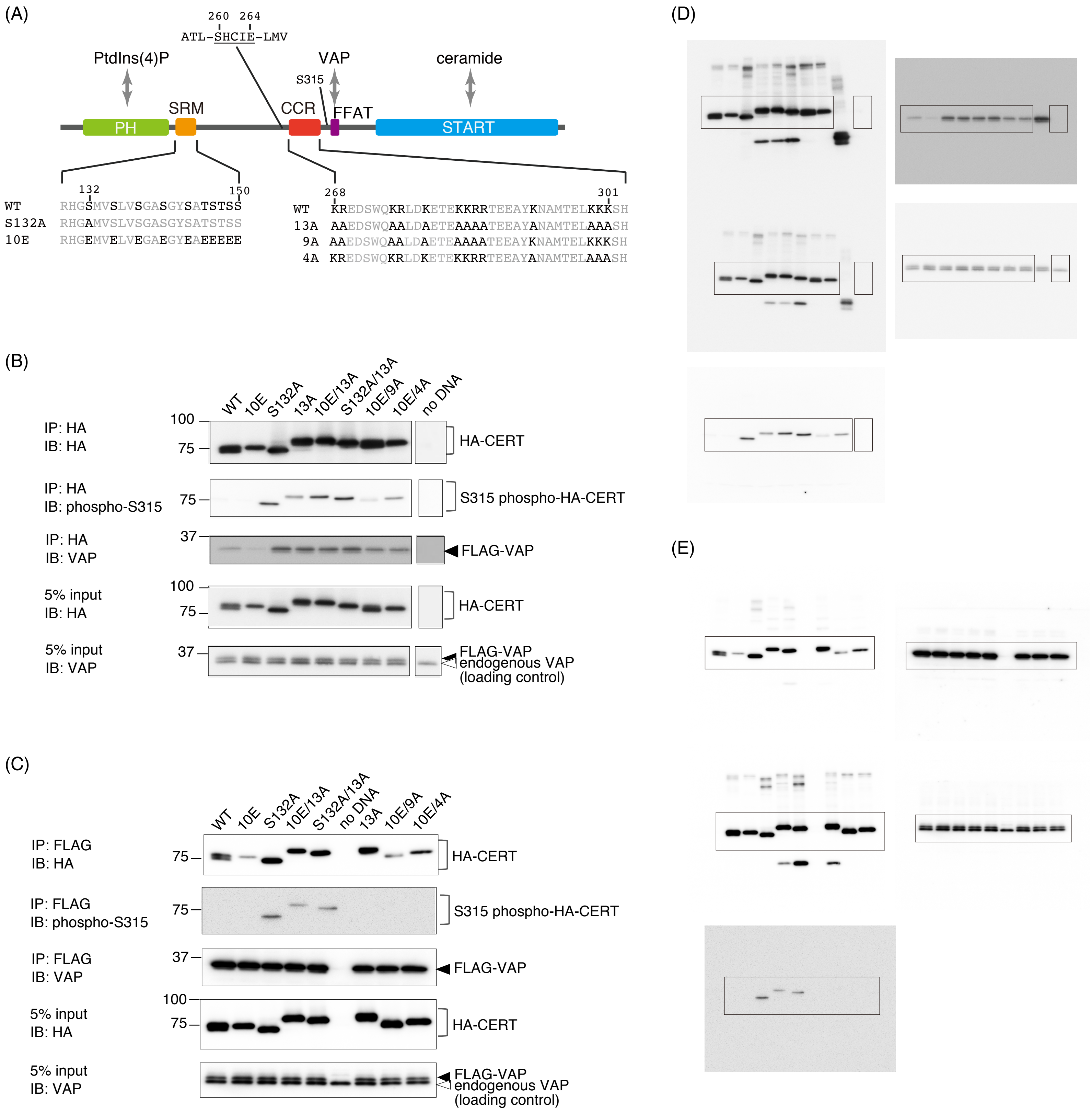

Figure S4. The 10E mutation-dependent repression of the serine 315 phosphorylation and VAP-binding of CERT were less reversed by the 9A or 4A mutation, compared with the 13A mutation. (A) Domains and motifs of CERT. Amino acid sequences are specified for the SRM and CCR in wild-type (WT) and mutants that were used in this study. Various constructs of HA-CERT (WT, 10E, S132A, 13A, 10E/13A, S132A/13A, 10E/9A, and 10E/4A) and FLAG-VAP-A were transiently expressed in HeLa CERT KO cells, cross-linked, lysed and co-immuno-precipitated (IP) using (B) anti-HA antibody- or (C) anti-FLAG M2-conjugated agarose gel. The immuno-precipitated and the 5% input samples were separated using SDS-PAGE and immunoblotted with anti-HA, anti-VAP-A, and anti-phospho-serine 315 antibodies. (D) and (E) represent uncropped western blotting images for (B) and (C) respectively.

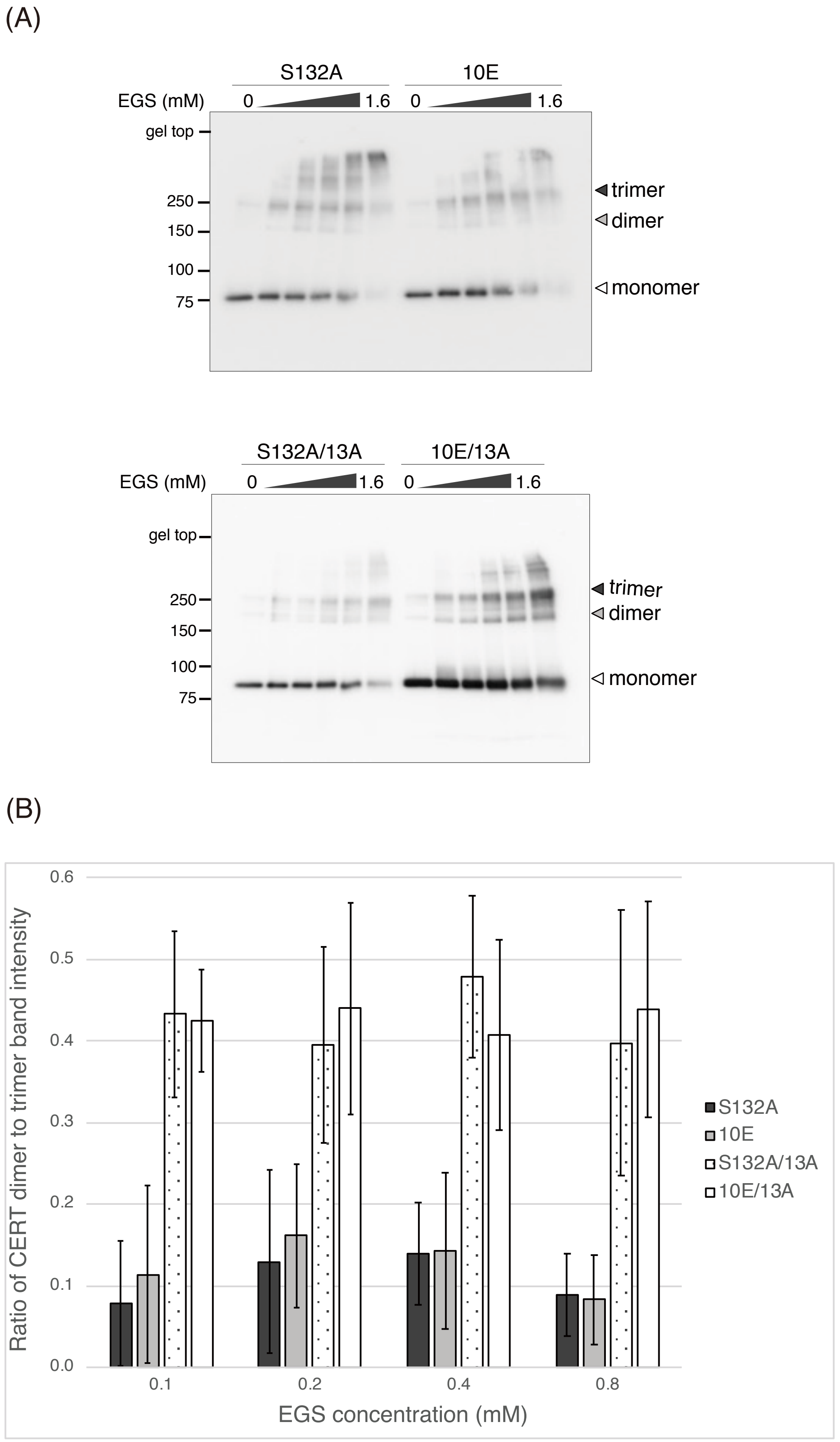

Figure S5. Cross-linking of purified recombinant CERT mutant proteins using various concentrations of EGS. (A) Purified recombinant CERT mutant protein samples were treated with 0, 0.1, 0.2, 0.4, 0.8, and 1.6 mM EGS and were analyzed by western blotting using anti-HA antibody. Uncropped images of the western blotting are shown. When the purified CERT preparations were treated with 1.6 mM EGS, larger oligomers or aggregates of CERT were produced, probably due to non-specific cross-linking. (B) The ratios of the dimer to trimer CERT bandintensity calculated from the experiment using 0.1 to 0.8 mM EGS in panel A are indicated as bar graphs.
